# Radiogenomic analysis of ultrasound phenotypic features coupled to proteomes predicts metastatic risk in primary prostate cancer

**DOI:** 10.1186/s12885-024-12028-9

**Published:** 2024-03-04

**Authors:** Qihuan Fu, Li Luo, Ruixia Hong, Hang Zhou, Xinzhi Xu, Yujie Feng, Kaifeng Huang, Yujie Wan, Ying Li, Jiaqi Gong, Xingyan Le, Xiu Liu, Na Wang, Jiangbei Yuan, Fang Li

**Affiliations:** 1 Department of Ultrasound, Chongqing Key Laboratory for Intelligent Oncology in Breast Cancer (iCQBC) , Chongqing University Cancer Hospital, 400030 Chongqing, China; 2 Department of Infection, Zhejiang Provincial People’s Hospital, 310014 Hangzhou, China

**Keywords:** Ultrasound phenotypic features, Quantitative proteomics, Primary prostate cancer, Metastatic risk, Correlation analysis

## Abstract

**Background:**

Primary prostate cancer with metastasis has a poor prognosis, so assessing its risk of metastasis is essential.

**Methods:**

This study combined comprehensive ultrasound features with tissue proteomic analysis to obtain biomarkers and practical diagnostic image features that signify prostate cancer metastasis.

**Results:**

In this study, 17 ultrasound image features of benign prostatic hyperplasia (BPH), primary prostate cancer without metastasis (PPCWOM), and primary prostate cancer with metastasis (PPCWM) were comprehensively analyzed and combined with the corresponding tissue proteome data to perform weighted gene co-expression network analysis (WGCNA), which resulted in two modules highly correlated with the ultrasound phenotype. We screened proteins with temporal expression trends based on the progression of the disease from BPH to PPCWOM and ultimately to PPCWM from two modules and obtained a protein that can promote prostate cancer metastasis. Subsequently, four ultrasound image features significantly associated with the metastatic biomarker HNRNPC (Heterogeneous nuclear ribonucleoprotein C) were identified by analyzing the correlation between the protein and ultrasound image features. The biomarker HNRNPC showed a significant difference in the five-year survival rate of prostate cancer patients (*p* < 0.0053). On the other hand, we validated the diagnostic efficiency of the four ultrasound image features in clinical data from 112 patients with PPCWOM and 150 patients with PPCWM, obtaining a combined diagnostic AUC of 0.904. In summary, using ultrasound imaging features for predicting whether prostate cancer is metastatic has many applications.

**Conclusion:**

The above study reveals noninvasive ultrasound image biomarkers and their underlying biological significance, which provide a basis for early diagnosis, treatment, and prognosis of primary prostate cancer with metastasis.

**Supplementary Information:**

The online version contains supplementary material available at 10.1186/s12885-024-12028-9.

## Introduction

Prostate cancer ranks second in incidence and fifth in mortality worldwide, making it one of the most prevalent malignant diseases among male cancer patients [1]. Approximately 15% of cases are metastatic at diagnosis [1]. Prostate cancer without metastasis can be effectively treated with surgical removal or radiation therapy, resulting in a 5-year survival rate of more than 90%. The 5-year survival rate decreases to 31% for prostate cancer with metastasis [1, 2]. Although the metastatic sites of prostate cancer exhibit diversity, the occurrence of metastasis depends on the biological behavior of the primary tumor [3]. Therefore, the assessment of prostate status before the biopsy is of great significance for accurate clinical diagnosis, formulation of optimal treatment, and evaluation of patient prognosis.

Ultrasound and multiparametric MRI (mp-MRI) are the primary imaging methods for assessing prostate cancer status. Although mpMRI has high tissue resolution, it has certain limitations due to its long scanning time, high cost, and contraindications [4]. Ultrasound, a low-cost, reproducible, and convenient imaging technology, is gradually gaining momentum in supplementing and replacing mpMRI [5, 6]. Multiple image features of ultrasound, such as grayscale, Doppler flow imaging, elastography, and contrast-enhanced ultrasound, are often used in varying degrees of combination for systematic scanning and biopsy of prostate patients. The study has used contrast-enhanced ultrasound parameters to predict bone metastasis [7]. Some studies have established a scoring system or machine learning model based on various ultrasonic image features and measurements, which are essential in identifying malignant prostate cancer and assessing prostate cancer risk [8–10]. However, studies still lack evaluating ultrasound image features to predict whether prostate cancer has metastasized. Using more comprehensive and straightforward ultrasound imaging features is of great practical significance for assessing prostate cancer with metastasis and a prerequisite for understanding the underlying biological functions behind the ultrasound phenotypic features.

Proteins play a critical role in completing biological functions. With the advancement of mass spectrometry, there has been a stream of proteomic studies centered on prostate cancer with lymph node or distant metastasis [11]. These studies reveal that the proteome of primary prostate cancer with metastasis exhibits more significant heterogeneity than primary prostate cancer [12]. As prostate cancer progresses to an advanced stage of metastasis, there is a more substantial alteration in the proteomic changes reflecting cell cycle and DNA damage [13, 14]. Several studies have identified protein markers that promote prostate cancer metastasis using proteomics techniques [15, 16]. However, molecular markers that accurately predict metastatic risk are still lacking. Also, these proteomic studies need to be matched with imaging and pathology to determine their clinical significance.

Radiogenomics combines the radiomics features with the molecular characteristics of tumor tissue, including genome, transcriptome, proteome, and metabolome, providing a new way to understand the biological functions behind the image [17]. The application of radiogenomics in studying ultrasonic phenotype and proteomics of prostate cancer with metastasis remains blank. This research extracted the ultrasonic phenotypic characteristics of patients with benign prostate cancer, prostate cancer without metastasis, and prostate cancer with metastasis. We then combined this data with proteomic information obtained from puncture biopsy tissues. Through correlation analysis, we aimed to uncover changes in proteomics associated with the ultrasonic phenotype of prostate cancer with metastasis and to investigate its potential biological significance.

## Materials and methods

### Ethical approval

All human tissues, clinical data, ultrasound images, and pathology results used in this study were approved by the Chongqing University Affiliated Tumor Hospital Ethics Review Committee (protocol code 2019 − 177 and date of approval 23 October 2019), and written consent was obtained from the patients. Patients’ names were anonymized according to ethical and legal standards.

### Clinical material and patient inclusion criteria

We collected clinical data, ultrasound images, and pathological results of prostate patients who underwent ultrasound-guided puncture at Chongqing University Affiliated Tumor Hospital from November 2018 to December 2022. Inclusion criteria were as follows: (1) Patients who underwent ultrasound-guided prostate targeted biopsy or radical prostatectomy within one week after contrast-enhanced ultrasound examination and obtained successful pathological results. (2) Ultrasound images, including grayscale, Doppler flow imaging, and contrast-enhanced ultrasound, were recorded and saved in DICOM format. Exclusion criteria were as follows: (1) Patients had a history of prostate disease or prostate surgery. (2) Patients were taking 5-alpha reductase inhibitors and/or endocrine therapy drugs for prostate cancer based on Pinto F et al.‘s report [18]. (3) The ultrasound images were missing or incomplete. (4) The pathological results and Gleason score were incomplete.

### Ultrasound scanning and imaging feature analysis

The patient’s prostate was sequentially scanned in the apex, middle, and base planes using the CANON Aplio 500 or i800 instrument equipped with a rectal cavity probe operating at a frequency of 5 ~ 9 MHz. Subsequently, the prostate was observed in the axial plane. All patients were diagnosed, and ultrasound doctors extracted ultrasound image features with more than three years of experience. Conventional ultrasound techniques employed in this process include grayscale and Doppler flow imaging, encompassing color Doppler flow imaging and power Doppler flow imaging. Imaging features observed and recorded in prostate cancer patients include envelope integrity, bilateral morphological symmetry, the demarcation between the inner and outer glands, features of suspected lesions (length, boundary, shape, echo pattern, and blood flow), suspected lesion state, suspected lesion location, suspected lesion distribution, and vessels penetrating between the inner and outer gland.

For contrast-enhanced ultrasound, 2.4 mL of Sonovar contrast agent was injected through the cubital vein, followed by 5 mL of normal saline. Upon initial injection of the contrast agent, the prostate was rapidly scanned sequentially. When suspicious lesions were detected, the probe was fixed, and another contrast agent injection was administered. Real-time monitoring of perfusion and regression patterns was carried out for approximately 5 min. A comparison was made between the suspected lesion and the surrounding tissue, as well as the corresponding site on the opposite side, to observe imaging features specific to prostate cancer patients. The contrast-enhanced Ultrasound imaging features include the demarcation between inner and outer glands, time to arrival compared to the normal region of the outer gland, peak enhancement compared to the normal region of the outer gland, time to arrival compared to the inner gland, and peak enhancement compared to the inner gland. All the collected ultrasound features mentioned above can be found in Supplementary Table 2.

### LC-MS/MS analysis

All tissue samples were collected from lesions with abnormal features by an experienced radiologist using an 18-G needle under ultrasound guidance. An automated tissue homogenizer (SPEX SamplePrep, MiniG) was employed to extract total protein from prostate tissues with SDS lysis solution (Beyotime, P0013G). The proteins (200 µg /sample) were digested for 16 h by trypsin. Q Exactive Orbitrap Mass Spectrometers equipped with UltiMate 3000 RSLCnano System (3 μm, 0.075 × 150 mm, Thermo Fisher) were used in this study. Previous articles described the liquid phase and mass spectrometry conditions [19, 20]. We identified and quantified proteins from the raw data of mass spectrometry using Proteome Discoverer (PD) 2.0 against the human RefSeq protein database. The search criteria are consistent with our previous reports [21–23].

### Construction of the weighted gene co-expression network

Weighted Gene Co-expression Network Analysis (WGCNA) analyzes gene expression patterns in multiple samples and clusters genes with similar expression patterns into modules that can be correlated with ultrasound phenotypes. We used the WGCNA software package to construct the gene co-expression network. To improve the reliability of the results, we set the minimum gene number to 30 and the sensitivity to 3.0 to construct a weighted co-expression network. Pearson correlation test was used to evaluate the correlation between each module and ultrasonic image features, and the key modules were analyzed.

### Survival analysis

Using data from the Human Protein Atlas website (https://www.proteinatlas.org/), we performed a Kaplan-Meier survival analysis by dividing prostate cancer patients into two groups based on the level of HNRNPC protein expression. We compared the survival outcomes of the two groups by log-rank tests (accession date: 2023-9-28).

### Receiver operating characteristic (ROC) curve analysis

The ROC analyses were presented using GraphPad Prism 8.3 software. A total of 262 patients who underwent ultrasound-guided puncture at Chongqing University Affiliated Tumor Hospital from November 2018 to December 2022 were included in this study.

### Protein-protein interaction network analysis

We used the STRING database (https://string-db.org/) for protein-protein interaction network analysis, setting the interaction score greater than 0.7. We used Cytoscape software to beautify the protein-protein interaction network.

### Bioinformatics analysis

The Gene ontology (GO) enrichment and Kyoto Encyclopedia of Genes and Genomes (KEGG) pathway analyses were performed using an online tool (The WebGestalt system, http://www.webgestalt.org/), setting significance level *p* < 0.05. The R software was used for other analyses in this study.

### Statistical analysis

We employed GraphPad Prism 8.3 software for statistical analysis and described the methods of analyzing significance in the corresponding figure legends.

## Results

### Description of the overall research program

**Fig. 1:**
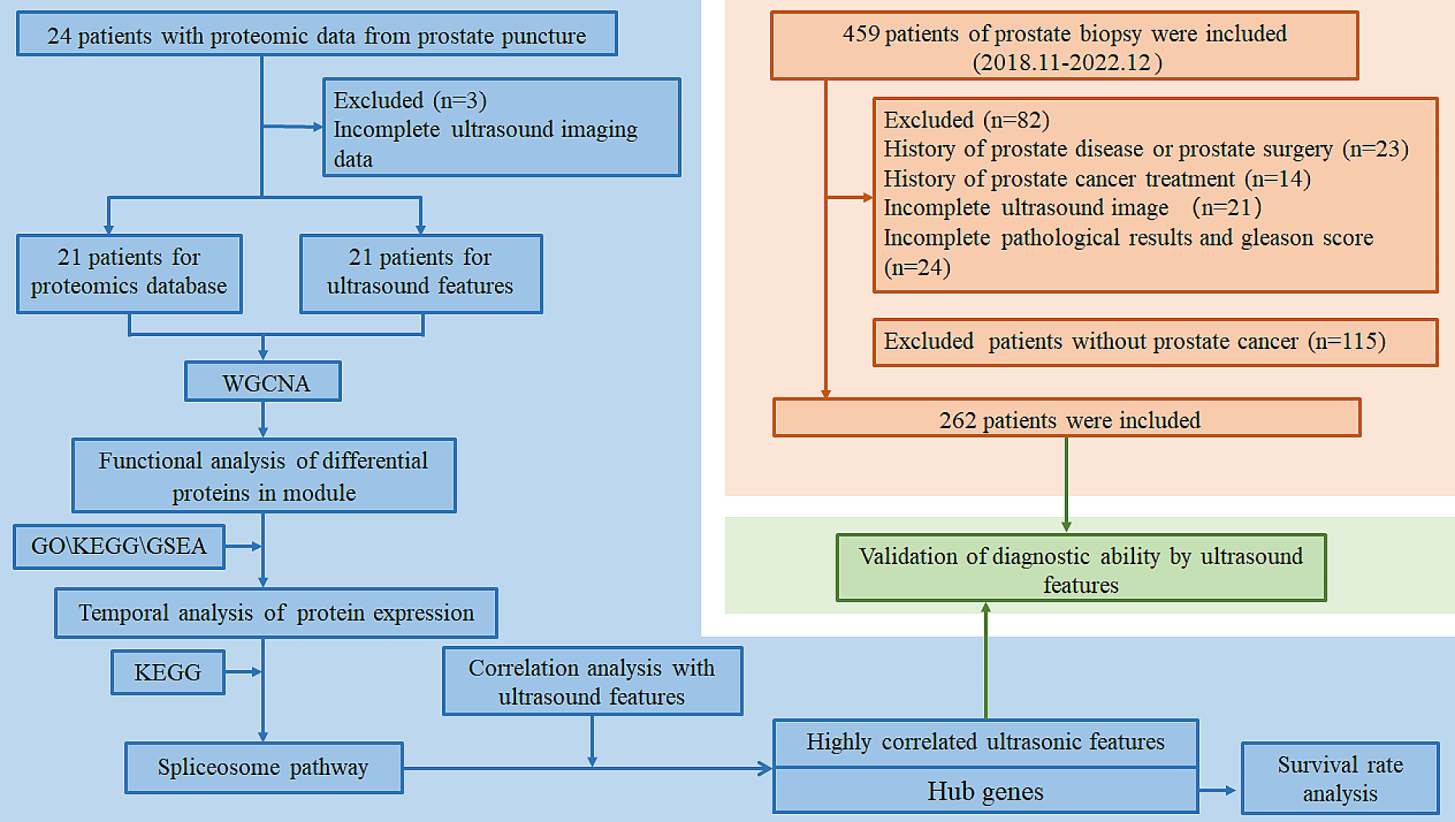
Flowchart of the study in this paper and inclusion criteria for clinical patients

To investigate the ability of ultrasound features combined with proteomics to detect primary prostate cancer with metastasis, we extracted 17 features from ultrasound images and established scoring rules (Supplemental Table 2). On the other hand, we collected prostate tissue from 24 patients for mass spectrometry analysis. However, with incomplete ultrasound imaging data from 3 patients, mass spectrometry data from 21 patients were used in subsequent research, including five patients with BPH, six with PPCWOM, and ten with PPCWOM. We showed the sample information, including the patient’s age (Supplemental Fig. 1A), the tumor-to-sample ratio (Supplemental Fig. 1B), the Gleason score (Supplemental Fig. 1C) and the free-to-total ratio of prostate-specific antigen (PSA, Supplemental Fig. 1D). We also displayed the H&E staining results of prostate tissue from 21 patients (Supplemental Fig. 2). Further, we presented clinical information (Supplemental Table 1), ultrasound feature scores (Supplemental Table 4), and proteins detected (Supplemental Table 3) from prostate tissue for these 21 patients. After a complex analytical process (Fig. 1), we developed an ultrasound-protein model that can be used to predict prostate cancer with metastasis. Finally, we validated this model using ultrasound characteristics and clinical data from 262 patients (Supplemental Table 5).

### Integrated analysis of ultrasound features and proteomic data

**Fig. 2:**
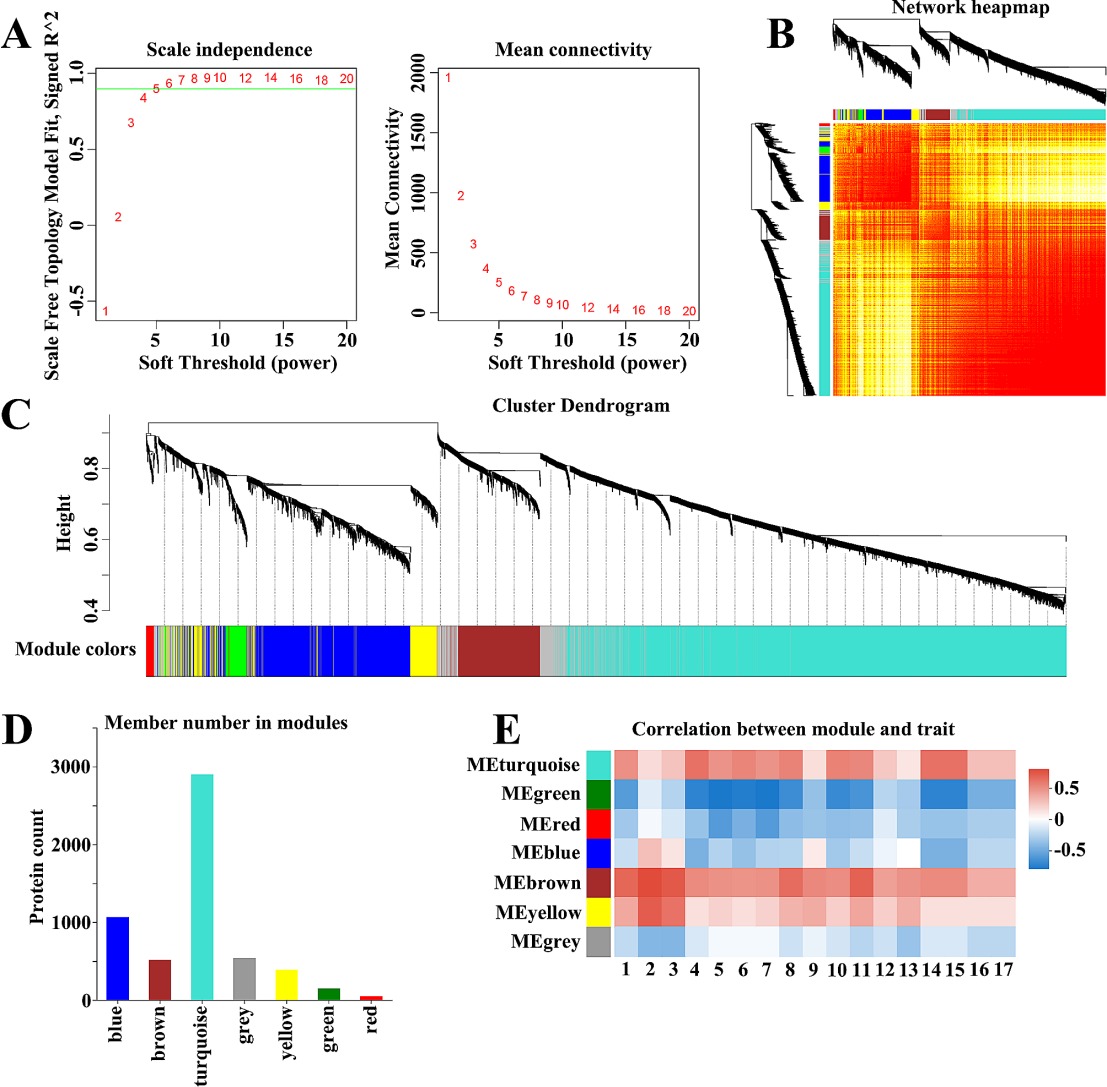
Identification of critical modules based on WGCNA. (**A**) Analyze the scale-free topology model fitting index (ft) and mean connectivity across a range of soft threshold powers (β) from 1 to 20. (**B**) Visualize protein networks using a network heatmap. Proteins are grouped into different modules through hierarchical clustering, and different colors represent different modules. (**C**) Clustering dendrogram: the top half represents a hierarchical clustering tree diagram of the proteins, while the bottom half corresponds to the protein modules. (**D**) Member number in modules. (**E**) Correlation between module and trait. 1 to 17 represent different ultrasound features. 1: Lesion length, 2: Envelope integrity, 3: Bilateral morphological symmetry, 4: The demarcation between the inner and outer glands, 5: Lesion boundary, 6: Shape of the lesion, 7: Echo pattern, 8: Lesion state, 9: Lesion location, 10: Lesion distribution, 11: Blood flow, 12: Penetrating vessel, 13: The demarcation between inner and outer glands after contrast-enhanced ultrasound, 14: Time to arrival compared to the normal region of the outer gland, 15: Peak enhancement compared to the normal region of the outer gland, 16: Time to arrival compared to the inner gland, 17: Peak enhancement compared to the inner gland

We constructed a hierarchical clustering tree using an unscaled co-expression network and topological overlap techniques based on ultrasound image features from 21 samples and their corresponding proteomic information. To create a scale-free network, we selected β = 5 as the soft-threshold power (Fig. 2A). Subsequently, we identified seven modules within the scale-free network (Fig. 2B and C). Each module was assigned a color, namely blue, brown, turquoise, grey, yellow, green, and red, with corresponding module protein counts of 1066, 514, 2900, 537, 387, 148, and 52, respectively (Fig. 2D). We have found a strong correlation between three modules and several ultrasound image features. Specifically, the turquoise module is highly correlated with feature 4 (R^2^ = 0.624, *p* = 0.0025), as well as feature 14 (R^2^ = 0.624, *p* = 0.0025) and feature 15 (R^2^ = 0.624, *p* = 0.0025). The brown module exhibits a strong correlation with feature 1 (R^2^ = 0.666, *p* = 0.000981), feature 2 (R^2^ = 0.774, *p* = 0.0000384), feature 3 (R^2^ = 0.719, *p* = 0.00024), feature 8 (R^2^ = 0.644, *p* = 0.00163), and feature 11 (R^2^ = 0.683, *p* = 0.000644). Similarly, the yellow module highly correlates with feature 2 (R^2^ = 0.706, *p* = 0.000349) and feature 3 (R^2^ = 0.622, *p* = 0.00261). Notably, the brown and turquoise modules showed a higher correlation with multiple ultrasound features, and the brown and turquoise modules were identified as hub modules (Fig. 2E).

### Screening of differential expression proteins and functional analysis in the turquoise module

**Fig. 3:**
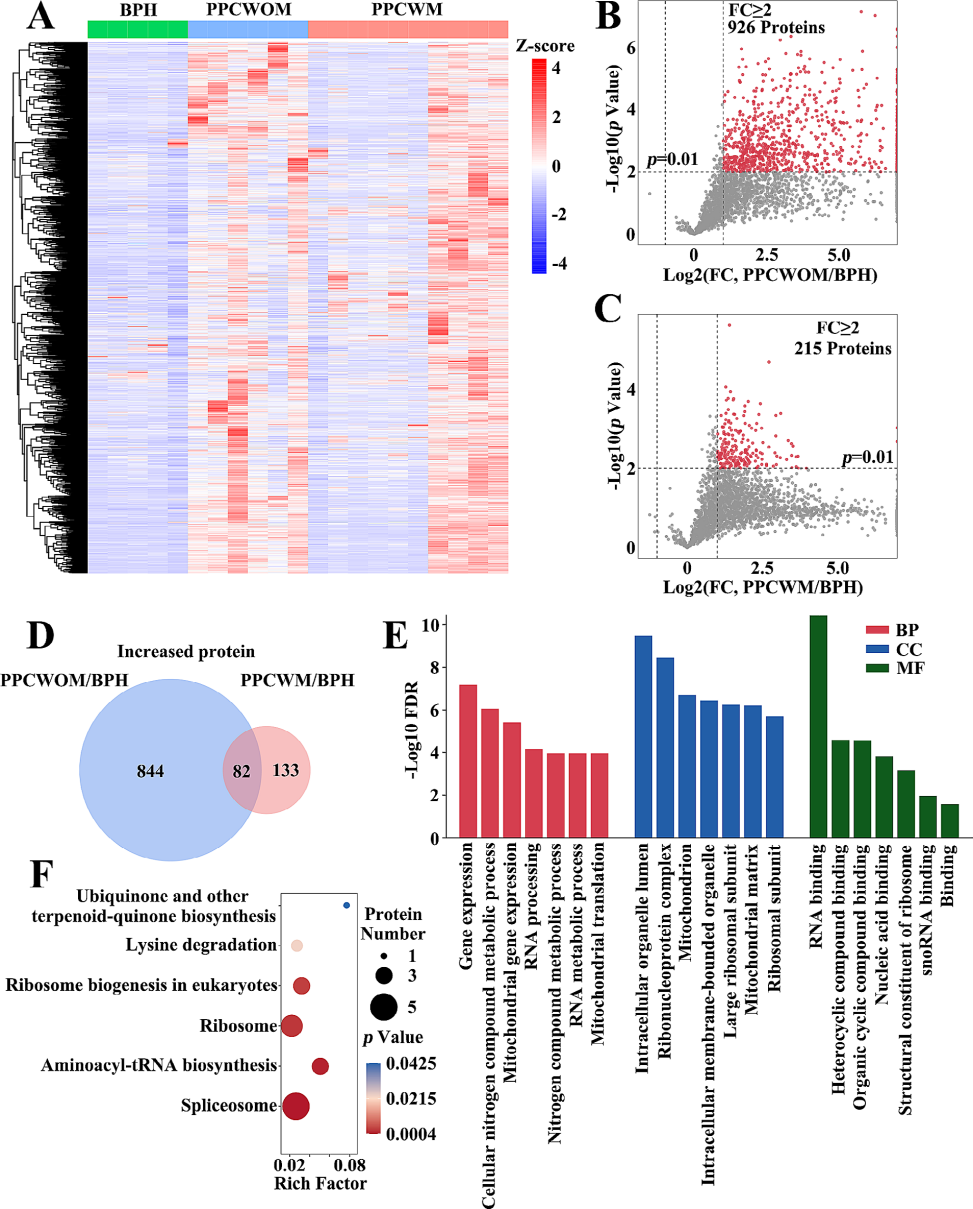
The protein expression and functional analysis of the turquoise module. (**A**) Heatmap depicting the expression of proteins in the turquoise module across samples of BPH (benign prostatic hyperplasia), PPCWOM (primary prostate cancer without metastasis), and PPCWM (primary prostate cancer with metastasis). (**B**) Differential expression proteins volcano plot between BPH and PPCWOM in the turquoise module. (**C**) Differential expression proteins volcano plot between BPH and PPCWM in the turquoise module. (**D**) Venn diagram shows 82 increased proteins shared in PPCWOM/BPH and PPCWM/BPH. (**E**) GO enrichment analysis of 82 differential expression proteins in biological process, molecular function, and cellular component. (**F**) KEGG pathway enrichment results for 82 differential expression proteins

We analyzed the expression patterns of the turquoise module in BPH, PPCWOM, and PPCWM (Fig. 3A). Based on a *p*-value < 0.01 and log2FC ≥ 2, we selected 926 differential expression proteins in PPCWOM/BPH (Fig. 3B), as well as 215 differential expression proteins in PPCWM/BPH (Fig. 3C). Furthermore, we identified 82 shared differential expression proteins in PPCWOM/BPH and PPCWM/BPH (Fig. 3D). We used GO enrichment analysis to reveal the functional distribution of these proteins in the Biological process (BP)、Cellular component (CC), and Molecular function (MF) (Fig. 3E). In BP, they were mainly involved in gene expression, cellular nitrogen compound metabolic process, mitochondrial gene expression, RNA processing, nitrogen compound metabolic process, RNA metabolic process, and mitochondrial translation. In CC, they were primarily associated with intracellular organelle lumen, ribonucleoprotein complex, mitochondrion, intracellular membrane-bounded organelle, large ribosomal subunit, mitochondrial matrix, and ribosomal subunit. In MF, they exhibited functionality related to RNA binding, heterocyclic compound binding, organic cyclic compound binding, nucleic acid binding, structural constituent of ribosome, snoRNA binding, and binding. Additionally, these proteins were significantly enriched in the KEGG pathway, such as spliceosome, aminoacyl-tRNA biosynthesis, ribosome, ribosome biogenesis in eukaryotes, lysine degradation, and ubiquinone and other terpenoid-quinone biosynthesis (Fig. 3F).

### Screening of differential expression proteins and functional analysis in the brown module

**Fig. 4:**
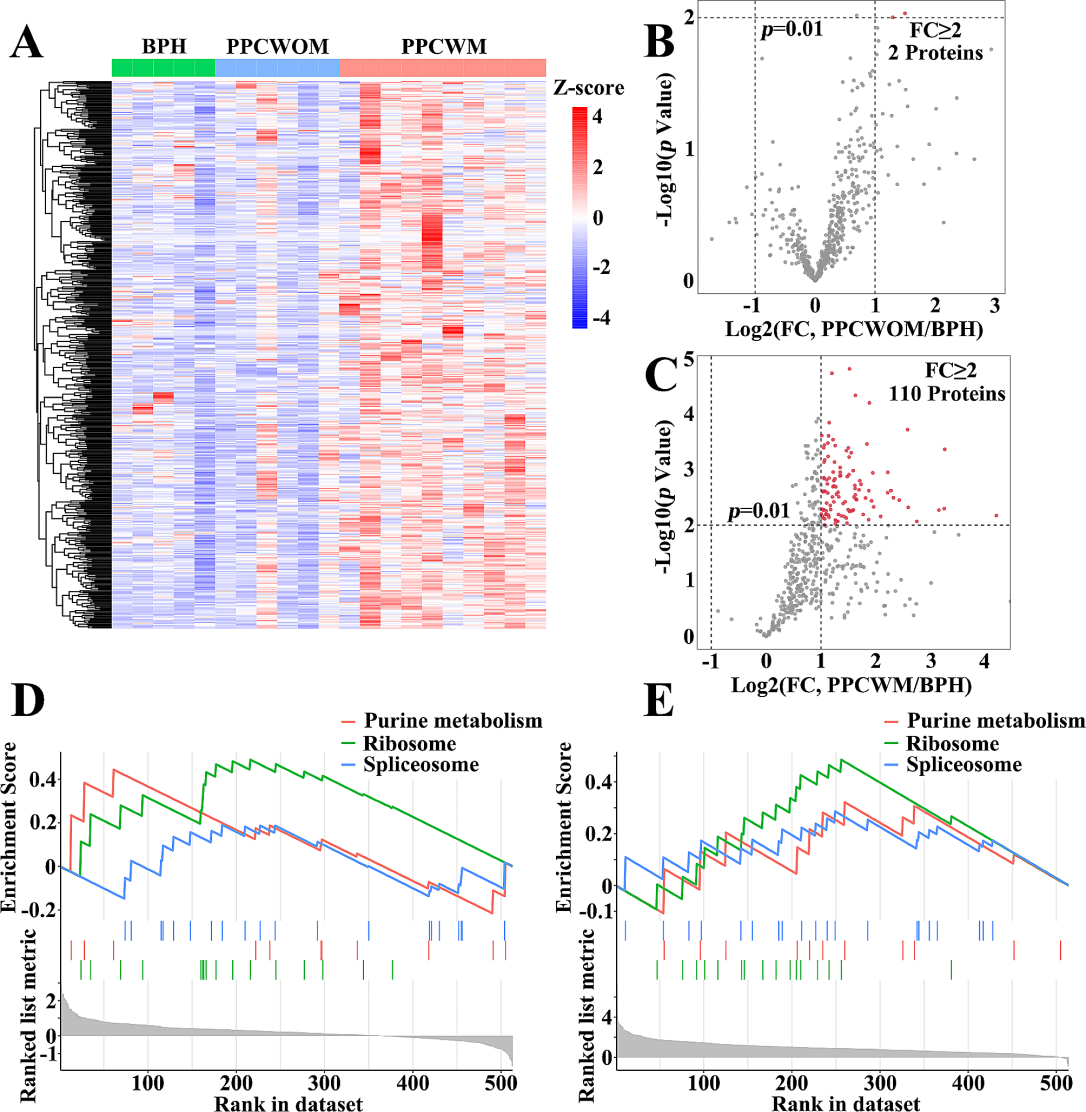
The protein expression and functional analysis of the brown module. (**A**) Heatmap depicting the expression of proteins in the brown module across samples of BPH, PPCWOM, and PPCWM (**B**) Differential expression proteins volcano plot between BPH and PPCWOM in the brown module. (**C**) Differential expression proteins volcano plot between BPH and PPCWM in the brown module. (**D**) Gene Set Enrichment Analysis (GSEA) for PPCWOM/BPH. (**E**) Gene Set Enrichment Analysis (GSEA) for PPCWM/BPH. Set significance level *p* less than 0.05

Next, we analyzed the expression patterns of the brown module in BPH, PPCWOM, and PPCWM (Fig. 4A). Based on a *p*-value < 0.01 and log2FC ≥ 2, we selected 2 differential expression proteins in PPCWOM/ BPH (Fig. 4B), as well as 110 differential expression proteins in BPH and PPCWM (Fig. 4C). Due to the limited number of differential expression proteins obtained through filtering, we conducted GSEA (Gene Set Enrichment Analysis) separately to analyze protein enrichment in PPCWOM/BPH and PPCWM/BPH. The results showed that the enriched pathways were mainly related to purine metabolism, ribosome, and spliceosome (Fig. 4D and E).

### Protein expression temporal analysis in the brown and turquoise modules

**Fig. 5:**
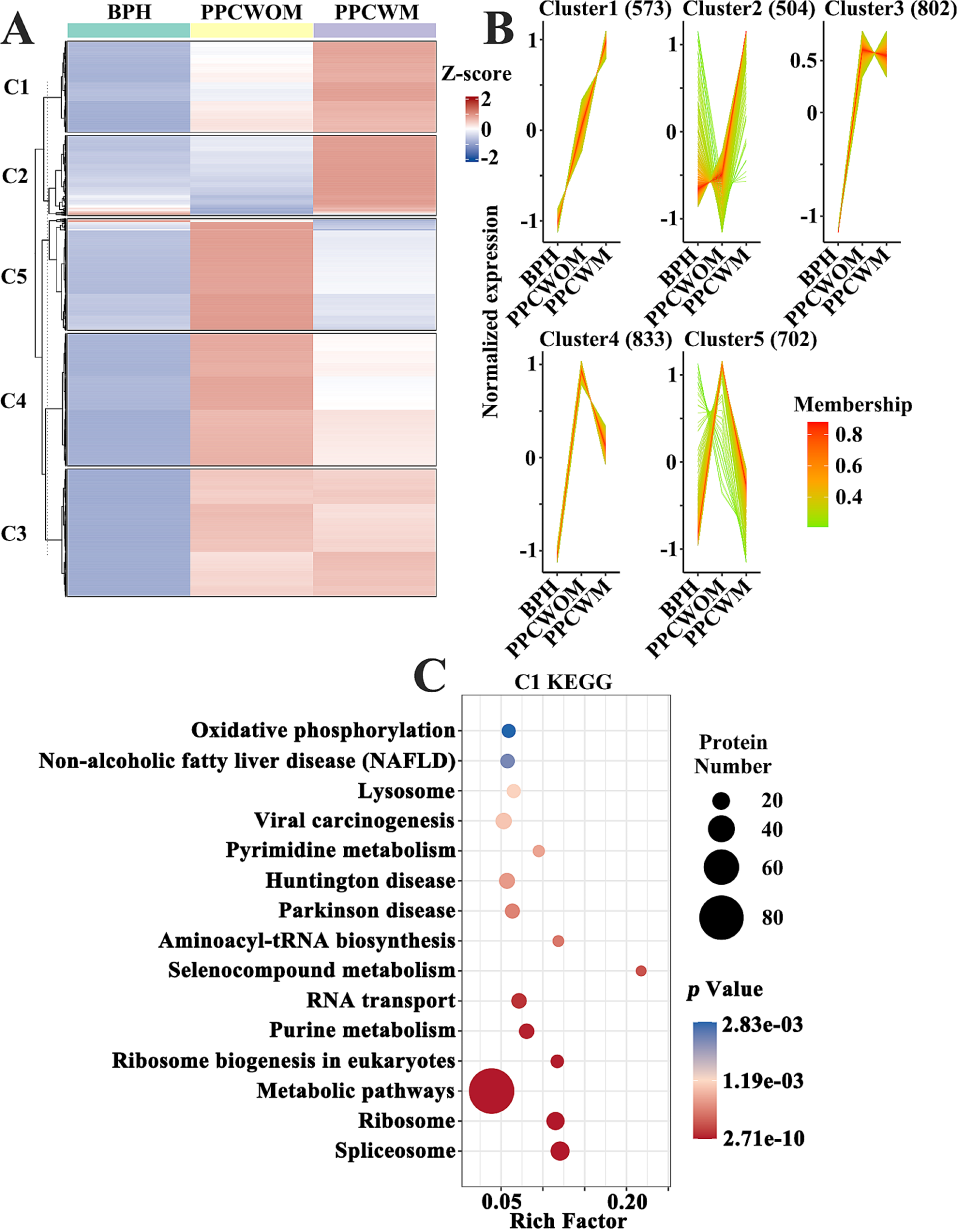
Proteins exhibiting temporal expression trends in the brown and turquoise modules were screened according to disease progression from BPH to PPCWOM and ultimately PPCWM. (**A**) The protein expression temporal analysis categorized expression trends into five classes, namely C1-C5. (**B**) The trend change line graphs demonstrate the expression alterations in the five trend classes. (**C**) KEGG pathway analysis for proteins of C1

We performed protein expression temporal analysis in the brown and turquoise modules to identify proteins with dynamic expression patterns from BPH to PPCWOM and eventually PPCWM (Fig. 5A). Five distinct expression trends were identified and categorized, with the C1 class showing a progressive increase in expression levels as the disease worsens (Fig. 5B). Furthermore, the KEGG pathway analysis for the C1 proteins revealed significant enrichment in the splicesome pathway (Fig. 5C).

### Ultrasound imaging features combined with proteomic analysis to predict prostate cancer prognosis and metastasis

**Fig. 6:**
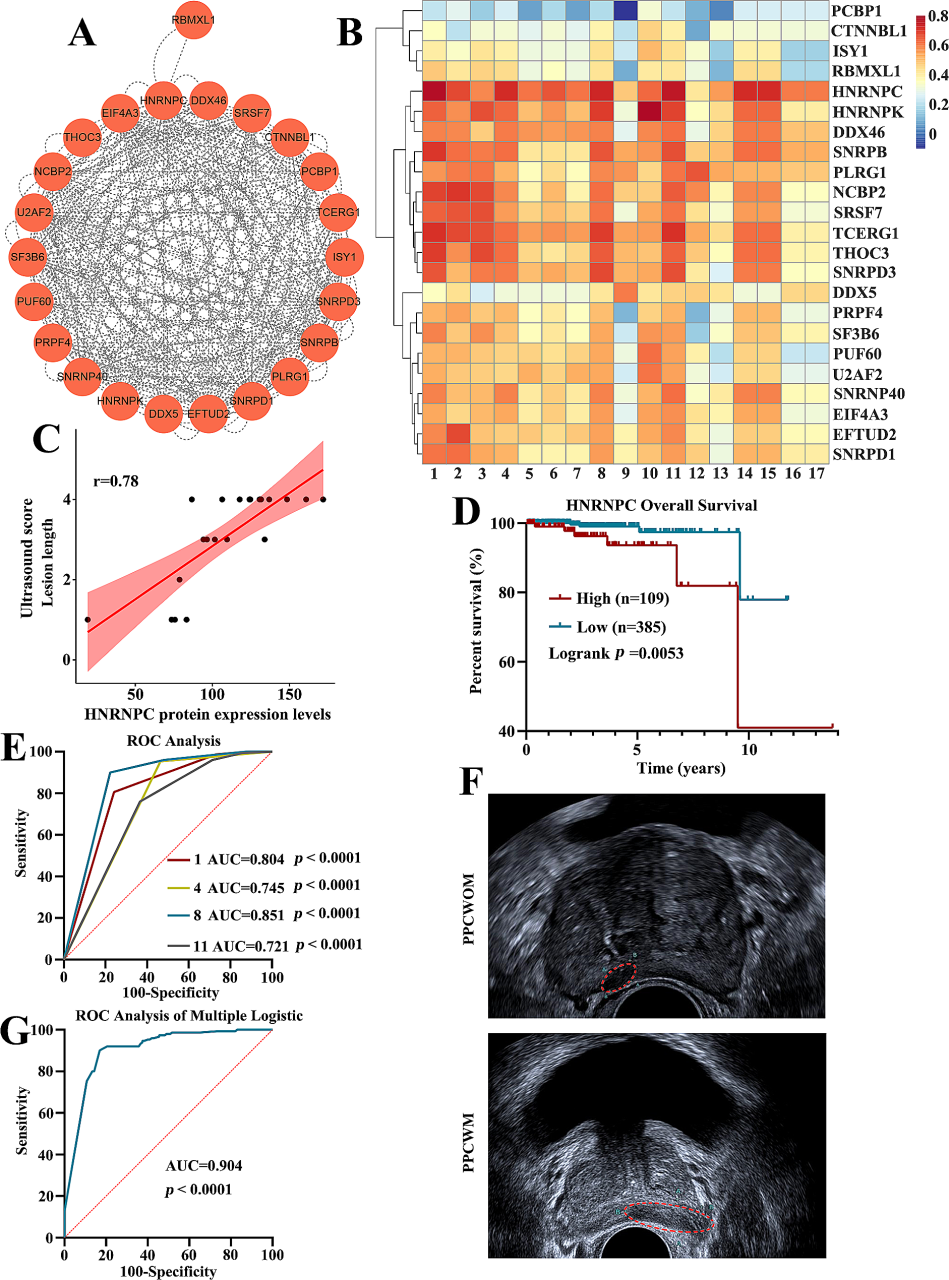
Ultrasound imaging features and HNRNPC protein correlation analysis predict prostate cancer prognosis and metastasis. (**A**) Protein-protein interaction (PPI) network of spliceosome pathway. (**B**) Heatmap of correlation between 17 ultrasound imaging features and proteins in the splicesome signaling pathway. (**C**) Scatterplot of correlation between HNRNPC expression level and lesion length. (**D**) Overall survival curve for HNRNPC protein. Differences were tested using the log-rank test. (**E**) ROC curves for ultrasound image features 1, 4, 8, and 11 in diagnosing prostate cancer metastasis. (**F**) Representative ultrasound images distinguish primary prostate cancer with metastasis and primary prostate cancer without metastasis by lesion length. (**G**) Combined diagnostic ROC curve for the four ultrasound image features (1, 4, 8, and 11). 1 to 17 represent different ultrasound features. 1: Lesion length, 2: Envelope integrity, 3: Bilateral morphological symmetry, 4: The demarcation between the inner and outer glands, 5: Lesion boundary, 6: Shape of the lesion, 7: Echo pattern, 8: Lesion state, 9: Lesion location, 10: Lesion distribution, 11: Blood flow, 12: Penetrating vessel, 13: The demarcation between inner and outer glands after contrast-enhanced ultrasound, 14: Time to arrival compared to the normal region of the outer gland, 15: Peak enhancement compared to the normal region of the outer gland, 16: Time to arrival compared to the inner gland, 17: Peak enhancement compared to the inner gland

We constructed protein-protein interaction networks from the splicesome pathway (Fig. 6A). Additionally, we performed a correlation analysis between these proteins and 17 ultrasound phenotypic features. The results showed a high correlation between HNRNPC protein and ultrasound features (Fig. 6B). The scatterplot revealed that the correlation between the HNRNPC protein expression level and the comprehensive ultrasound features reached 0.78 (Supplementary Fig. 3). Among them, HNRNPC showed the highest correlation with lesion length, with a similarity coefficient of 0.78 (Fig. 6C). The correlation of HNRNPC with the demarcation between the inner and outer glands, lesion state, blood flow, time to arrival compared to the normal region of the outer gland, and peak enhancement compared to the normal region of the outer gland also reached 0.72, 0.73, 0.76, 0.72 and 0.72, respectively (Supplementary Fig. 4). Further analysis revealed a significant difference in the five-year survival rate of prostate cancer patients based on HNRNPC expression (*p* < 0.0053) (Fig. 6D).

Finally, 112 patients with PPCWOM and 150 patients with PPCWM were included in this study. The four features with the highest correlation with HNRNPC expression levels (feature 1, lesion length; feature 4, the demarcation between the inner and outer glands; feature 8, lesion state; feature 11, blood flow) were evaluated using Receiver Operating Characteristics (ROC) curves to identify the diagnostic capabilities for metastatic prostate cancer patients. The results showed that features 1, 4, 8, and 11 exhibited high diagnostic capabilities. The Area Under the Curve (AUC) values for these features were 0.804, 0.745, 0.851, and 0.721, respectively (*p* < 0.0001) (Fig. 6E). Also, we showed these four ultrasound features representative images in PPCWOM and PPCWM patients (Fig. 6F and Supplementary Fig. 5). When these four ultrasound imaging features were combined, the diagnostic ability was further enhanced, with an AUC of 0.904 (*p* < 0.0001) (Fig. 6G). The above results suggest that combining four ultrasound imaging features may improve the accuracy of diagnosing prostate cancer with metastasis.

## Discussion

In this study, we utilized comprehensive and practical ultrasound image features integrated with proteomics to explore the biological functions performed by proteomics behind ultrasound images. We also demonstrated the temporal expression changes of proteins through the progression from benign prostatic hyperplasia to localized prostate cancer to prostate cancer with metastasis. Proteins with temporal expression trends were correlated with ultrasound image features to obtain protein markers contributing to the development of prostate cancer and ultrasound image features with diagnostic capability for primary prostate cancer with metastasis.

Current studies have utilized grayscale, color/power Doppler imaging, and quantitative ultrasonographic parameters to analyze echo and flow differences to assess the metastaticity of prostate tumors and non-quantitative ultrasound phenotypic features for prostate cancer risk stratification [7, 8]. These studies set the stage for our evaluation of prostate tumors with metastasis. Usually, qualitative characteristics are more accessible to measure and observe than quantitative parameters, and they are also suitable for generalization to primary hospitals. Therefore, the 17 ultrasound phenotypic features of prostate cancer we selected were mainly qualitative.

Further combining proteomics and clinical data, we screened four ultrasound features with high diagnostic power for prostate cancer with metastasis. Currently, most medical images are analyzed through artificial intelligence. However, the black box of artificial intelligence analysis remains a mystery to our understanding of medical images, and the resulting set of features is complex to interpret clinically [24]. Through our KEGG-based proteomic analysis, we have observed a significant enrichment of proteins associated with ultrasound features in the spliceosome and ribosome. Ribosomal abnormalities can culminate in aberrant or excessive protein expression, perturbing genes implicated in cell proliferation, metabolism, and angiogenesis [25]. Analogously, spliceosomal aberrations can disrupt the equilibrium or fidelity of mRNA isoforms, with attendant consequences for the coding or non-coding functionality of critical genes and the regulation of crucial signaling cascades and cellular processes [26, 27]. Notably, these alterations, especially those stemming from metastasis and angiogenesis, have the potential to be discerned via ultrasound imaging. The association of ultrasound phenotypic features with proteomics makes it possible to identify the biological causes that drive changes in ultrasound images.

We screened and identified HNRNPC, a critical gene that promotes prostate cancer development, through ultrasound image features in combination with proteomics. This gene showed the highest correlation with four ultrasound image features. HNRNPC, one of the earliest members of the HNRNP family, regulates alternative splicing and affects mRNA maturation, stability, translocation, and translation [28]. Analysis of the GEO dataset showed that HNRNPC was significantly upregulated in prostate cancer samples compared to normal samples [29]. The expression of HNRNPC in primary prostate cancer with metastasis or castration-resistant prostate cancer samples was even higher than that in localized prostate cancer [29]. In addition, HNRNPC has been shown to promote the proliferation, migration, and invasion of prostate cancer cells [29]. The above results support that ultrasound image features combined with proteomic analysis can screen for proteins that drive prostate cancer progression.

This study also has some limitations. Firstly, the sample size incorporated in this research is inadequate, necessitating a larger sample size to enhance the precision of the study. Secondly, recognizing ultrasound image features relies on the expertise of medical professionals. Despite our physicians possessing over three years of clinical experience and adhering to standardized image acquisition protocols, certain features require further discernment. Moreover, this study simplifies the feature collection process, thereby minimizing the inclusion of quantitative features, leading to the inevitability of ambiguous features.

## Conclusions

This study used comprehensive and practical ultrasound image features and proteomics for WGCNA analysis. We identified the relevant biomarkers and associated ultrasonic image features to promote prostate cancer with metastasis, providing a new understanding of the tumor biological function behind the ultrasound phenotype of prostate cancer.

### Electronic supplementary material

Below is the link to the electronic supplementary material.

Supplementary Material 1

Supplementary Material 2

Supplementary Material 3

Supplementary Material 4

Supplementary Material 5

Supplementary Material 6

Supplementary Material 7

Supplementary Material 8

Supplementary Material 9

Supplementary Material 10

Supplementary Material 11

## Data Availability

The mass spectrometry proteomics data were deposited in the ProteomeXchange Consortium (http://proteomecentral.proteomexchange.org) with the dataset identifier PXD028211.

## Abbreviations

LC-MS/MS: Liquid chromatography-mass spectrometry/mass spectrometry
WGCNA: Weighted gene co-expression network analysis
BPH: Benign prostatic hyperplasia
PPCWOM: Primary prostate cancer without metastatic
PPCWM: Primary prostate cancer with metastatic
GSEA: Gene set enrichment analysis
ROC: Receiver operating characteristic
