# Supplementary figures and images for: Radiogenomic analysis of ultrasound phenotypic features coupled to proteomes predicts metastatic risk in primary prostate cancer
Source: BMC Cancer. 2024 Mar 4;24:290. doi: 10.1186/s12885-024-12028-9 (PMC10913270; doi:10.1186/s12885-024-12028-9)

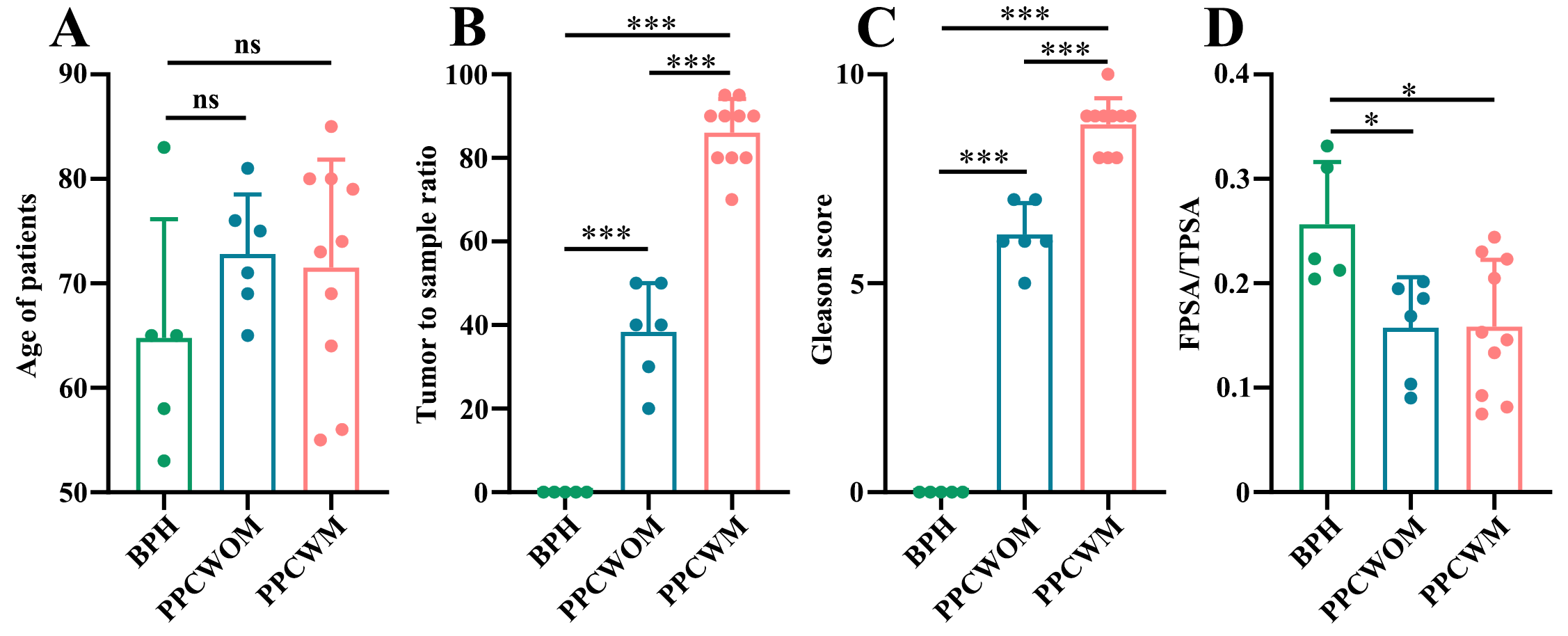

Supplement: Supplementary file 1 — Supplementary Material 1 [file 12885_2024_12028_MOESM1_ESM.png]

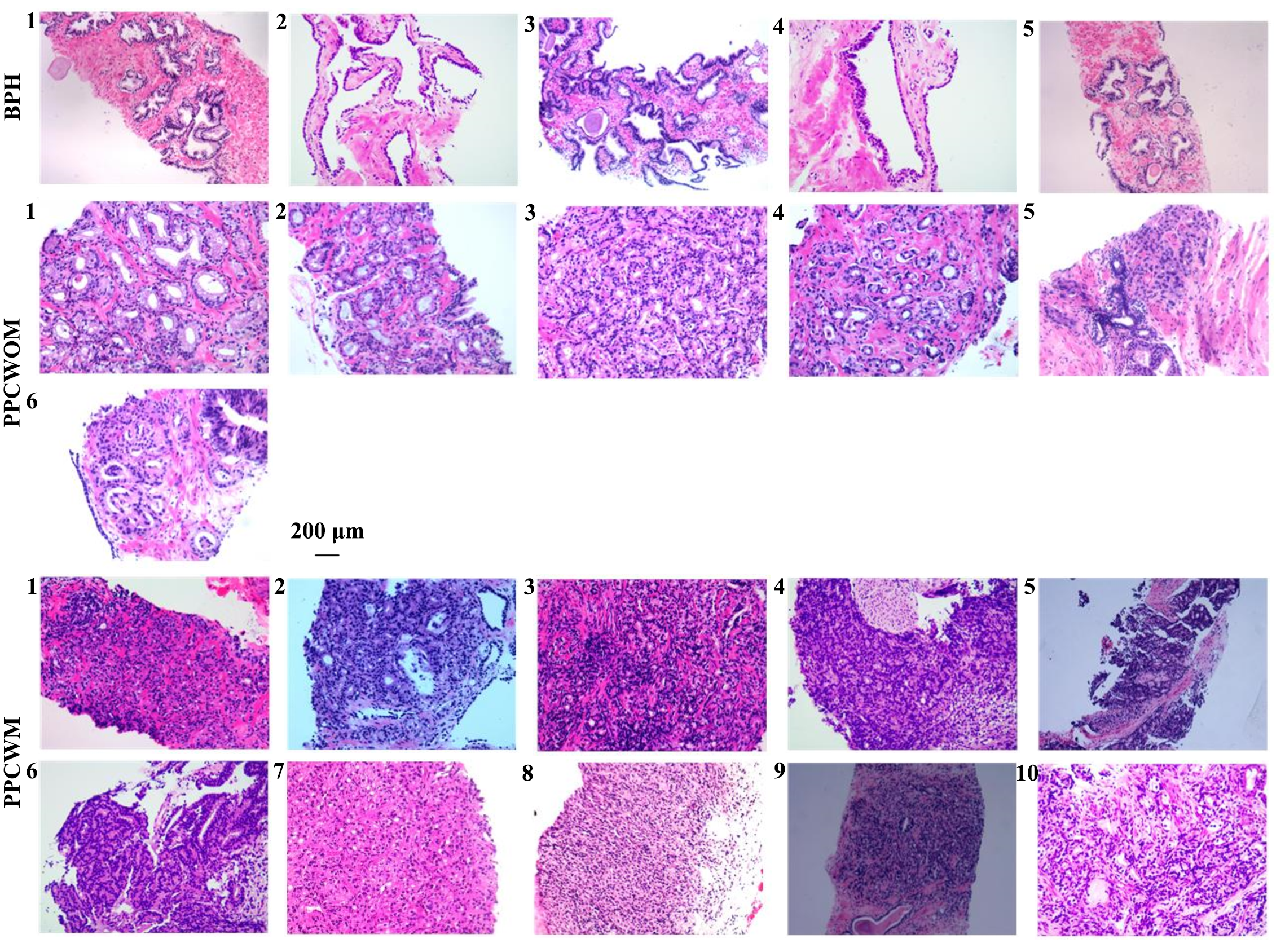

Supplement: Supplementary file 2 — Supplementary Material 2 [file 12885_2024_12028_MOESM2_ESM.png]

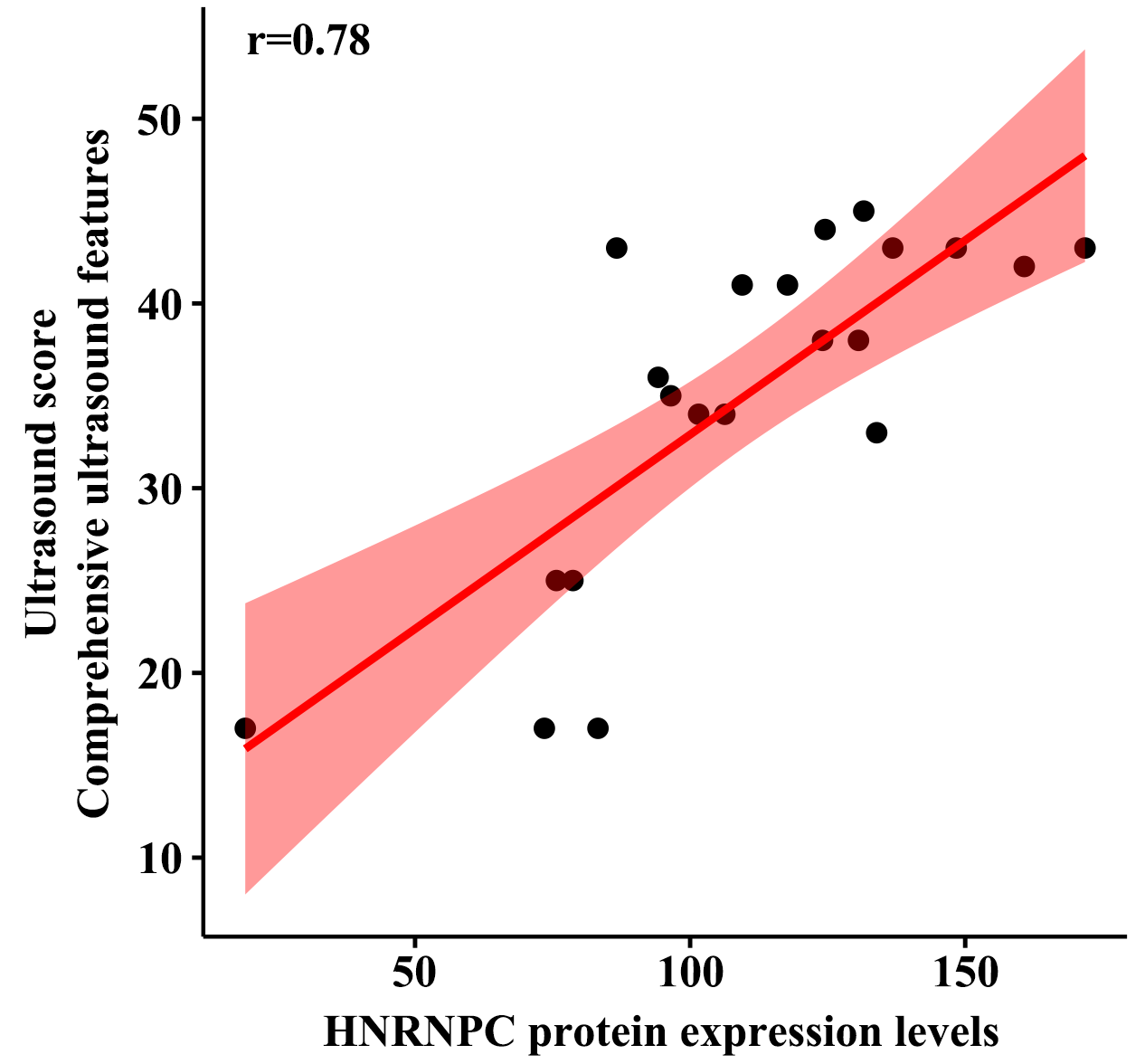

Supplement: Supplementary file 3 — Supplementary Material 3 [file 12885_2024_12028_MOESM3_ESM.png]

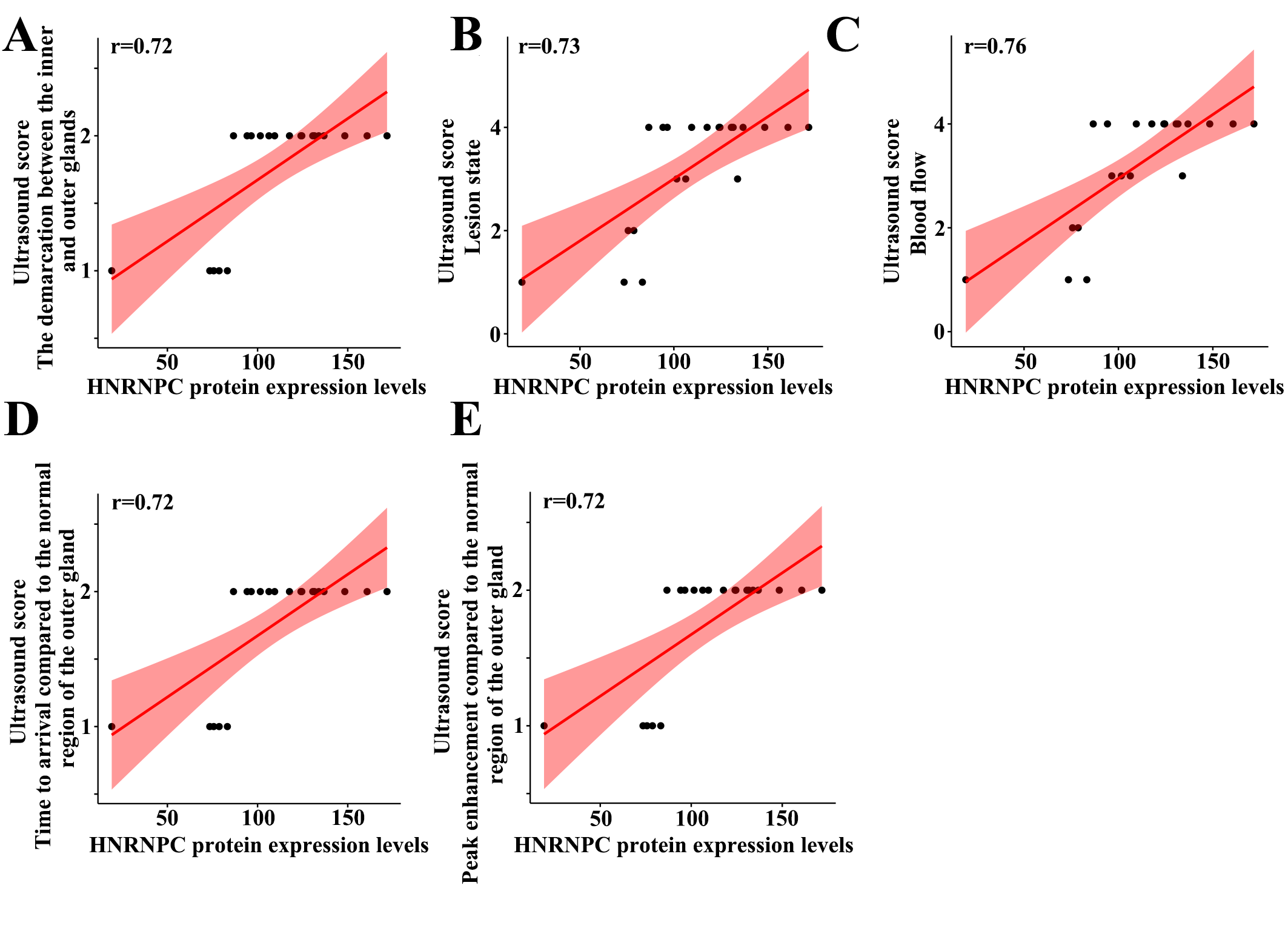

Supplement: Supplementary file 4 — Supplementary Material 4 [file 12885_2024_12028_MOESM4_ESM.png]

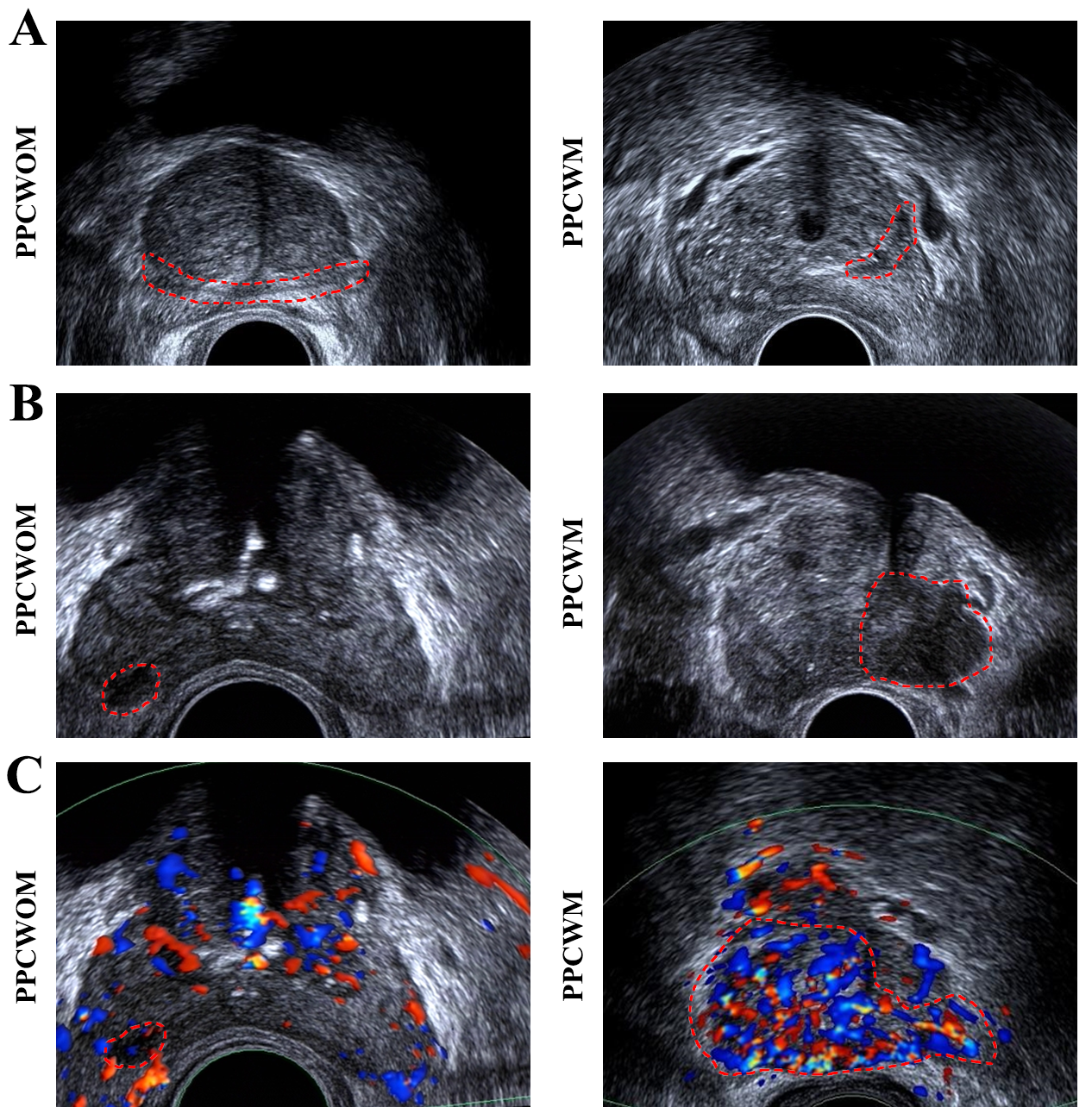

Supplement: Supplementary file 5 — Supplementary Material 5 [file 12885_2024_12028_MOESM5_ESM.png]
