# Supplementary material for: Radiogenomic analysis of ultrasound phenotypic features coupled to proteomes predicts metastatic risk in primary prostate cancer
Source: BMC Cancer. 2024 Mar 4;24:290. doi: 10.1186/s12885-024-12028-9 (PMC10913270; doi:10.1186/s12885-024-12028-9)
Supplement: Supplementary file 11 — Supplementary Material 11 [file 12885_2024_12028_MOESM11_ESM.docx]

**Supplemental information**

**Supplemental Figure 1.** Information of Prostate tissues. (A) The average age of patients. (B) The tumor-to-sample ratio. (C) The Gleason score for patients. (D) The free-to-total ratio of PSA.

**Supplemental Figure 2.** The H&E staining results of prostate tissue from 21 patients

**Supplemental Figure 3.** Scatterplot of correlation between HNRNPC expression level and comprehensive ultrasound features. The scores for the comprehensive ultrasound features were obtained by accumulating the 17 ultrasound feature scores.

**Supplemental Figure 4.** Scatterplot of correlation between HNRNPC expression level and (A) the demarcation between the inner and outer glands, (B) lesion state, (C) blood flow, (D) time to arrival compared to the normal region of the outer gland, and (E) peak enhancement compared to the normal region of the outer gland.

**Supplemental Figure 5.** Representative ultrasound images distinguish primary prostate cancer with metastasis and primary prostate cancer without metastasis by (A) the demarcation between the inner and outer glands, (B) lesion state, and (C) blood flow.

**Supplemental Table 1.** Clinical information of 21 patients whose punctured prostate tissues were used for mass spectrometry analysis.

**Supplemental Table 2.** Scoring rules for ultrasound imaging parameters.

**Supplemental Table 3.** Proteins were identified in the prostate tissues of 21 patients.

**Supplemental Table 4.** Ultrasound parameter score of 21 patients.

**Supplemental Table 5.** Clinical information and ultrasound parameter scores for 262 patients treated at Chongqing University Affiliated Tumor Hospital from November 2018 to December 2022.
